# New parameterization of air-sea exchange coefficients and its impact on intensity prediction under major tropical cyclones

**DOI:** 10.3389/fmars.2022.1046511

**Published:** 2022-11-16

**Authors:** Woojeong Lee, Sung-Hun Kim, Il-Ju Moon, Michael M. Bell, Isaac Ginis

**Affiliations:** 1 Forecast Research Department, National Institute of Meteorological Sciences, Jeju, South Korea; 2 Graduate Program in Marine Meteorology/Typhoon Research Center, Jeju National University, Jeju, South Korea; 3 Korea Institute of Ocean Science and Technology, Busan, South Korea; 4 Department of Atmospheric Science, Colorado State University, Fort Collins, CO, United States; 5 Graduate School of Oceanography, University of Rhode Island, Narragansett, RI, United States

**Keywords:** air-sea exchange coefficients, lifetime maximum intensity, maximum potential intensity, numerical modeling, tropical cyclone intensity predictions

## Abstract

Understanding and quantifying air-sea exchanges of enthalpy and momentum fluxes are crucial for the advanced prediction of tropical cyclone (TC) intensity. Here, we present a new parameterization of air-sea fluxes at extreme wind speeds from 40 m s^−1^ to 75 m s^−1^, which covers the range of major TCs. Our approach assumes that the TC can reach its maximum potential intensity (MPI) if there are no influences of external forces such as vertical wind shear or other environmental constraints.This method can estimate the ratio of the enthalpy and momentum exchange coefficient (*C*_k_/*C*_d_) under the most intense TCs without direct flux measurements. The estimation showed that *C*_k_/*C*_d_ increases with wind speed at extreme winds above 40 m s^−1^. Two types of surface layer schemes of the Hurricane Weather and Research Forecast (HWRF) were designed based on the wind speed dependency of the *C*_k_/*C*_d_ found at high winds: (i) an increase of *C*_k_/*C*_d_ based on decreasing *C*_d_ (Cd_DC) and (ii) an increase of *C*_k_/*C*_d_ based on increasing *C*_k_ (Ck_IC). The modified surface layer schemes were compared to the original HWRF scheme (using nearly fixed *C*_d_ and *C*_k_ at extreme winds; CTRL) through idealized experiments and real-case predictions. The idealized experiments showed that Cd_DC reduced frictional dissipation in the air-sea interface as well as significantly reduced sea surface cooling, making the TC stronger than other schemes. As a result, Cd_DC reduced the mean absolute error and negative bias by 15.0% (21.0%) and 19.1% (32.0%), respectively, for all lead times of Hurricane Irma in 2017 (Typhoon Mangkhut in 2018) compared to CTRL. This result suggests that new parameterization of *C*_k_/*C*_d_ with decreasing *C*_d_ at high winds can help improve TC intensity prediction, which currently suffers from underestimating the intensity of the strongest TCs.

## Introduction

Prediction of the tracks of tropical cyclones (TCs) has significantly improved over the last decades, but there has been less progress in intensity prediction despite the considerable advancement in technologies for TC-related physics, computational power, numerical modeling, and observations during the same period (DeMaria et al., 2014; Soloviev et al., 2014). TC intensity prediction is challenging because the various environmental effects, inner core dynamics, and underlying surface forcings involved in the intensity change are not entirely understood (Elsberry et al., 2013). In particular, the unresolved physics of the momentum and enthalpy transfer between the atmosphere and the ocean at extreme wind speeds are crucial factors that hamper accurate TC intensity prediction (Moon et al., 2004; Moon et al., 2007).

Figure S1 shows the bias of the HWRF in predicting the 10 m maximum wind speed (MWS) for 2016-2020 in the North Atlantic (NA), the eastern North Pacific (ENP) and the western North Pacific (WNP) TCs, on the basis of 82,821 predictions. The HWRF is an atmosphere-ocean coupled model customized for the hurricane or tropical storm application (Biswas et al., 2018); it is a real-time TC forecasting system operational at the National Centers for Environmental Prediction (NCEP) since the 2007 TC season. Overall, a negative bias (underestimation) is evident above 40 m s^−1^, significantly increasing with MWS (Figure S1). It reaches −20 m s^−1^ for intense TCs with an MWS above 80 m s^−1^. This significant bias may be partly attributed to the insufficient horizontal grid resolution of the model (Bender and Ginis, 1999). Another possible reason is the inadequate representation of some physical processes, including the air-sea momentum and enthalpy exchanges at high wind speeds.

TC intensity depends strongly on the coefficients of the transfers of momentum (*C*_d_) and enthalpy (*C*_k_) between the ocean and the atmospheric boundary layer (Ooyama, 1969; Rosenthal, 1971; Emanuel, 1986; Bryan, 2012; Green and Zhang, 2013; Green and Zhang, 2014; Zhang and Emanuel, 2016). In particular, the theoretical MWS of a TC depends on the ratio of the enthalpy coefficient to the momentum exchange coefficient—(*C*_k_/*C*_d_)^1/2^—in the high–wind speed core of the storm (Emanuel, 1986). Estimating the most accurate *C*_k_/*C*_d_ and its behavior at high wind speeds is essential for accurate TC intensity prediction (Green and Zhang, 2013; Soloviev et al., 2014).

The perceived importance of the dependence of the TC intensity on the *C*_k_/*C*_d_ has led to considerable effort to measure the exchange coefficients at hurricane-strength wind speeds (DeCosmo et al., 1996; Black et al., 2007; French et al., 2007; Zhang et al., 2008; Haus et al., 2010; Bell et al., 2012; Hsu et al., 2019; Curcic and Haus, 2020; Richter et al., 2021). However, these observations deliver less accuracy at high winds. For exchange coefficient estimates based on the mean profile data obtained from dropsondes in TCs, the accuracies are known to be 50% and 200% for *C*_d_ and *C*_k_, respectively (Richter et al., 2016). Likewise, laboratory estimations of *C*_d_ and *C*_k_ have limitations because conditions of field observations such as wave age, wavelength, spray, and fetch cannot be considered realistically. These limitations make the determination of air-sea exchanges at extreme wind speeds and their wind dependency difficult (Richter et al., 2016).

In this study, we propose an alternative method in which *C*_k_/*C*_d_ at very high wind speeds is indirectly estimated by matching the observed lifetime maximum intensity (LMI) of a TC with the theoretical maximum potential intensity (MPI). This method is based on the assumption that the LMI can reach the MPI if there are no negative influences on the maximum achievable intensity (Emanuel, 1995; Bister and Emanuel, 1998). The equation is

(1) $$MPI^{2}=\frac{C_{k}}{C_{d}}\frac{T_{sea}-T_{o}}{T_{o}}\left(k^{*}-k\right)≅LMI^{2}$$

(2) $$\frac{C_{k}}{C_{d}}=LMI^{2}{\left(\frac{T_{sea}-T_{o}}{T_{o}}\left(k^{⋆}-k\right)\right)}^{-1}$$

where *T*_sea_ is the sea surface temperature (SST) under a storm, *T*_o_ is the TC outflow temperature determined by the atmospheric vertical profile, k* is the saturation enthalpy of the sea surface, and *k* is the surface enthalpy in the TC environment.

The relationship between MPI and LMI (Equation (1)) can only be applied under limited TC conditions. For example, the relationship is invalid under the following conditions: (1) The storm moves quickly from a region with a high potential intensity to one with low potential intensity. In this case, the actual LMI can exceed the theoretical MPI because a storm needs time to adjust to its new environment. (2) The storm faces unfavorable environmental conditions, such as a strong vertical wind shear (VWS), or is influenced by the land. In this case, the actual LMI cannot approach the theoretical MPI. (3) The storm is weak. In this case, the actual LMI cannot match the theoretical MPI, given that the structure of a weak storm cannot sensitively respond to a changing environment. Therefore, by selecting only the TCs that satisfy these strict conditions, we can use the new approach to quantitatively estimate the ratio of the exchange coefficients for intense TCs (Equation (2)).

The objectives of this study are (i) to investigate the behavior of *C*_k_/*C*_d_, as a function of wind speed, under severe winds, and (ii) to examine whether the new air-sea exchange flux parameterization based on current results can contribute to improving TC intensity prediction. We designed a numerical experiment using the state-of-the-art HWRF model to achieve the second objective and applied it to idealized and real hurricane cases. Section 2 describes the data and methods used in this study. In Section 3, we show the estimated Ck/Cd results and compare them with the findings of other methods. Section 4 shows the result of TC prediction using modified exchange coefficients in HWRF. The conclusions and discussions are provided in Section 5.

## Data and methods

The LMI is estimated using the TC position and intensity obtained from the best track data of the Joint Typhoon Warning Center (JTWC) for the WNP, and the National Hurricane Center (NHC) for the NA and the ENP. For storms that achieved their LMI more than once, the actual LMI is chosen when the storm maintains its intensity for longer. Since the LMI of TCs can reach the theoretical MPI under limited conditions, we select only TCs that satisfy the following conditions: (1) The storm should have sufficient time to adjust to the new environment as it moves; i.e., the storm translation speed should not exceed 7 m s^−1^. (2) The storm should keep quasi-steady conditions at the LMI stage; i.e., it should maintain its strength for at least 12 h after reaching the LMI. (3) The environmental conditions along the storm track should not significantly change for at least 12 h after the storm achieves its LMI. (4) The representative dynamic and thermodynamic factors related to the TC intensity, VWS, and SST should not be unfavorable for storm development. Specifically, the average VWS within a 300 km radius should be lower than 10 m s^−1^, and the SST should be higher than 26°C. (5) The storm should have an intensity of at least Category 2 (above 40 m s^−1^). (6) The storm should be unaffected by land; i.e., there are no landmasses within a 300 km radius of the storm center. From the 2,255 TCs that occurred in the NA, ENP, and WNP basins between 1980 and 2015, the period when the TC intensity can be accurately estimated using geostationary satellites, 84 TCs satisfied the above conditions (Table S1, Figure S2).

The MPI is calculated using Emanuel’s potential intensity program (‘pcmin’ code) based on Equation 1, in which a scaling factor (VREDUC) of 0.8 is used to reduce the gradient wind to the surface wind. For the MPI calculation, the atmospheric input is obtained from the daily atmospheric temperature and humidity profile data from the Modern-Era Retrospective Analysis for Research and Applications (MERRA) dataset in a 1.5° × 1.5° longitude-latitude grid. The SST under the storm (*T*_sea_) is obtained from oceanic reanalysis data from the NCEP Global Ocean Data Assimilation System (GODAS), which provides pentad ocean subsurface temperatures at 40 geometric depth levels in a 1.5° × 0.333° longitude-latitude grid for 1980 to present. The depth-averaged temperatures (*T*_80_) from the surface to a depth of 80 m are calculated by averaging the GODAS data from the surface to the 80 m depth after the ocean temperature is interpolated to a 1 m depth interval. The MPI, including enthalpy (*k* and k*) at the LMI location, is calculated by averaging the values within a 300 km radius of the storm center using prestorm conditions (3 days before LMI) (Lin et al., 2013). To test the sensitivity of the SST data to the *C*_k_/*C*_d_ calculation, we calculate additional MPIs using Group for High-Resolution Sea Surface Temperature (GHRSST) data, which are produced daily at the Canadian Meteorological Centre with a spatial resolution of 0.2° × 0.2°. Since the GHRSST data are only from September 1991, the MPIs are derived only for the 38 TC cases that satisfy the previously described conditions from 1991 to 2015.

Numerical prediction experiments for the idealized and real TC cases are conducted using the HWRF to test the new air-sea exchange flux parameterization. This study uses the 2017 HWRF version, which has a 3 km spatial resolution. Further details regarding the input data and model setup are described in Biswas et al. (2018). The International Best Track Archive for Climate Stewardship (IBTrACS), produced by the NHC and the JTWC, is used to verify the TC forecasts (Knapp et al., 2010).

## New parameterization of *C*_k_/*C*_d_

**FIGURE 1:**
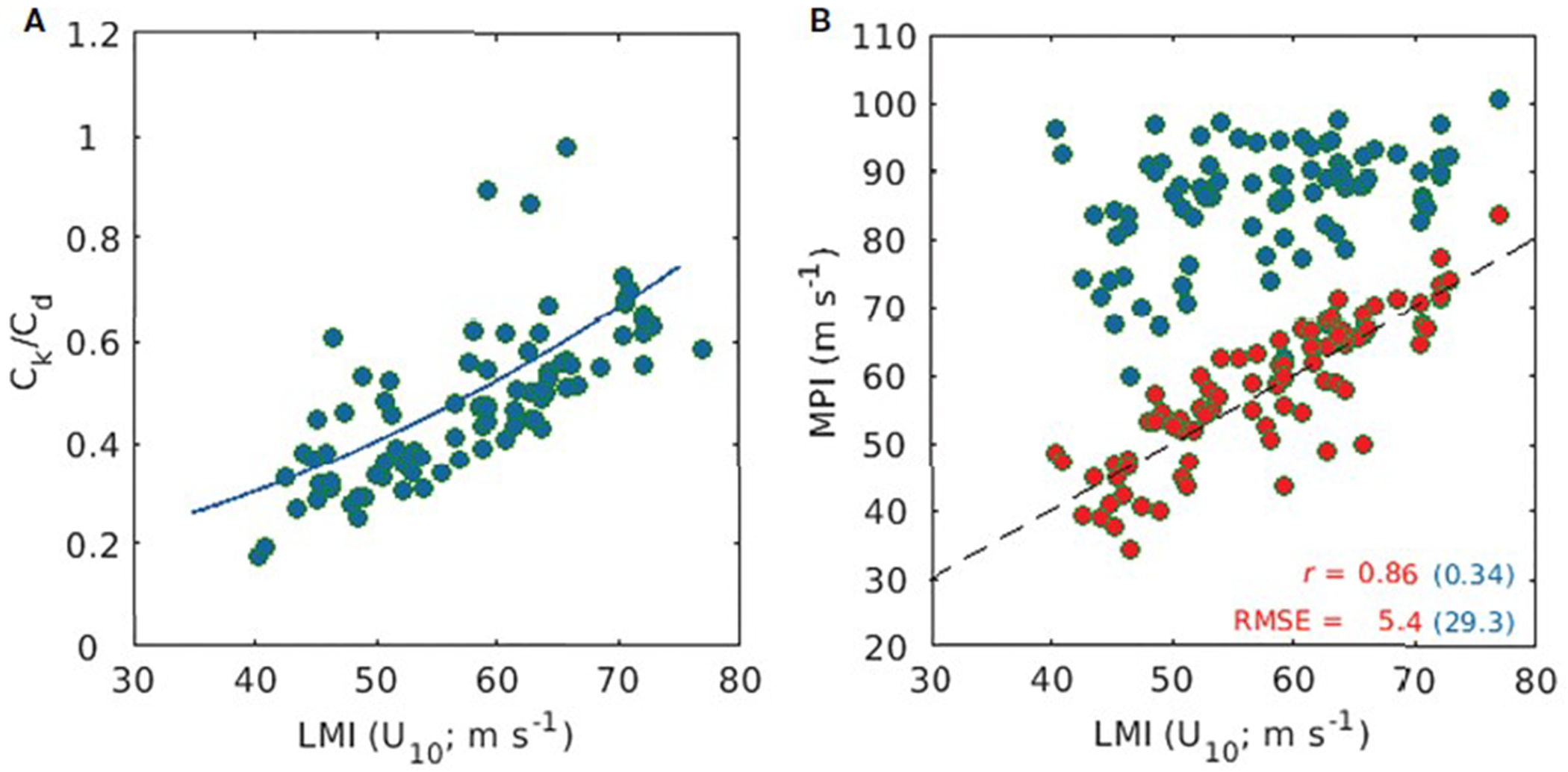
Scatter plot of *C*_k_/*C*_d_ and theoretical MPI against LMI (i.e., MWS at 10 m, *U*_10_) for major TCs. **(A)** Scatter diagram of *C*_k_/*C*_d_ and its linear fit (blue line, 1.1013e-04 *U*_10_^2^ + 0.1281) onto LMI. **(B)** Comparison of MPI calculated using unity (traditional way, blue dots) and linear fit (red dots) estimated by present approach for *C*_k_/*C*_d_. Correlation coefficients (*r*) and root mean square errors (RMSEs, m s^−1^) are shown in the lower right corner.

**FIGURE 2:**
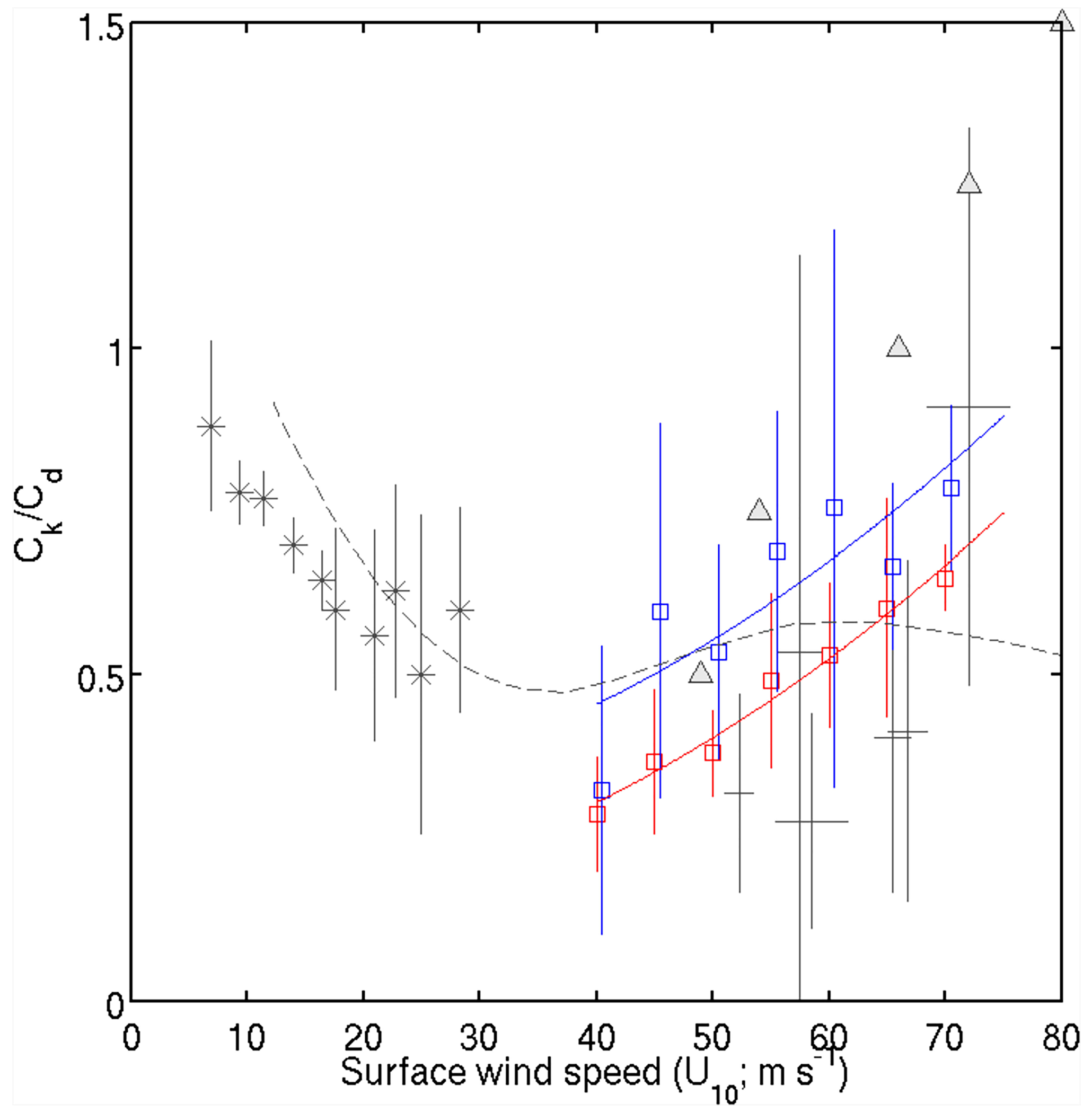
Dependence of *C*_k_/*C*_d_ on *U*_10_ based on prestorm SST and depth-averaged temperatures compared with previous studies. The red and blue squares indicate the mean values of *C*_k_/*C*_d_ within each 5 m s^−1^ interval of wind speed, estimated using prestorm SST and depth-averaged temperatures (*T*_80_), respectively. The color lines and error bars indicate linear fits and one standard deviation in the bins (the linear fit of the blue line is 1.0943e-04 *U*_10_^2^ + 0.2799). The dashed gray lines indicate data adapted from Soloviev et al. (2014), and the gray triangles indicate data adapted from Emanuel (1995). The mean and 95% confidence intervals of diverse laboratory and measurement results (DeCosmo et al., 1996; Zhang et al., 2008; Haus et al., 2010; Bell et al., 2012) are shown in gray asterisks and solid lines.

Figure 1A shows a scatter plot of *C*_k_/*C*_d_ as a function of LMI, which is estimated using Equation (2) for the 84 selected TCs. The estimated *C*_k_/*C*_d_ ranges from 0.2 to 1 for wind speeds of 40–75 m s^−1^, and it clearly increases with the MWS. Based on these results, we estimate the wind dependency of the ratio using a regression function (Figure 1A) and apply this, instead of the traditionally used constant *C*_k_/*C*_d_ (unity), to Equation 1. As a result, the correlation between the observed LMI and the theoretical MPI significantly increases from 0.34 to 0.86. The error decreases from 29.3 m s^−1^ to 5.4 m s^−1^ (refer to the blue and red dots shown in Figure 1B, respectively). Therefore, the increasing *C*_k_/*C*_d_ with wind speed at strong winds above 40 m s^−1^ may help estimate a realistic intensity of intense TCs (this possibility is examined in the next section). A comparison of our mean *C*_k_/*C*_d_ values (red symbol and line in Figure 2) with those in earlier studies, especially at wind speeds above 40 m s^−1^, shows that our results are generally within the range of Bell et al. (2012) but much smaller than those of Emanuel (1995). Figure S3 shows the prestorm SST–based *C*_k_/*C*_d_ obtained using GHRSST and GODAS. The similar pattern between the two datasets suggests that the present method is not highly sensitive to the SST data selection and the considered analysis period.

In the original MPI method, *T*_sea_ is calculated using the prestorm SST (Emanuel, 1988; Emanuel, 1995; Holland, 1997; Wang and Wu, 2004), which does not include the contribution of TC-induced SST cooling. The MPI in Figure 1 is also estimated using the prestorm SST. A recent study suggested that prestorm depth-averaged (averaged from the surface mixing depth down to the expected TC-induced mixing depth) ocean temperatures are more appropriate for calculating the MPI than the prestorm SST (Lin et al., 2013). This is based on *in situ* air-deployed ocean-atmosphere measurement pairs collected during the Impact of Typhoons on the Ocean in the Pacific (ITOP) program. In particular, the depth-averaged temperature (*T*_80_) from the surface to a depth of 80 m has been identified as the most appropriate index related to TC intensity under a wide range of major TC conditions (Price, 2009; Lin et al., 2013), although the TC-induced mixing depth also depends on the TC translation speed, size, and intensity and the upper ocean thermal structure (Price et al., 1994; Lin et al., 2009; Price, 2009).

Figure 2 compares the *C*_k_/*C*_d_ estimated using the prestorm SST and *T*_80_ (blue symbol and line). The most distinct discrepancy between the two results is an overall increase in the mean *C*_k_/*C*_d_ values and their standard deviations when *T*_80_ is used. This result implies that a higher *C*_k_/*C*_d_ value is required for the storm to reach a certain intensity when the effect of storm-induced surface cooling on the MPI calculation is considered. It also suggests that numerical experiments without negative ocean feedback may lead to a different conclusion about the behavior and magnitude of *C*_k_/*C*_d_ (Montgomery et al., 2010; Bryan, 2012; Green and Zhang, 2013; Li et al., 2016). A large increase in the standard deviation of *T*_80_ can be explained by the fact that the depth-averaged temperature can vary significantly due to different factors, such as the mixing depth and subsurface structure. A comparison of the two mean *C*_k_/*C*_d_ values with those in earlier studies at wind speeds above 40 m s^−1^ shows that our results are generally higher than those of Bell et al. (2012) for all covered wind speeds except the 72.5 m s^−1^ bin, where the values are similar. At wind speeds of 40–55 m s^−1^, the *T*_80_-based *C*_k_/*C*_d_ regression line generally agrees with that of Soloviev et al. (2014) in terms of the positive slope of *C*_k_/*C*_d_ (blue and dashed lines in Figure 2). However, at wind speeds above 55 m s^−1^, Soloviev et al.’s ratio decreases, while our calculated ratio increases with wind speed.

The continuously increasing trend of *C*_k_/*C*_d_ at high winds is qualitatively consistent with previous results (Emanuel, 1995; Andreas, 2011; Bao et al., 2011; Bell et al., 2012; Richter and Stern, 2014). The increasing trend of *C*_k_/*C*_d_ at high winds is possibly due to increasing *C*_k_ (Mueller and Veron, 2014) and/or decreasing *C*_d_ (Powell et al., 2003; Jarosz et al., 2007; Vickery et al., 2009; Soloviev et al., 2014). Some previous studies attribute this tendency to the effect of sea spray (Andreas, 2011; Bao et al., 2011). As wind speed increases, the mean droplet size and the mass flux of sea spray increase. The *C*_k_/*C*_d_ trend of continuously increasing at extreme wind speeds above 40 m s^−1^ may support the importance of spray-mediated air-sea enthalpy and momentum fluxes under TCs. However, the precise mechanisms responsible for this trend remain unknown.

The *C*_k_/*C*_d_ presented here may have significant implications for improving operational TC prediction, which currently tends to underestimate major hurricanes’ intensity severely. The HWRF uses *C*_k_/*C*_d_ parameterization of the curved-fitting to available field measurements from recent observations (Biswas et al., 2018). The *C*_k_/*C*_d_ value used in the HWRF may be too low at wind speeds above 60 m s^−1^. In fact, the *C*_k_/*C*_d_ from our *T*_80_-based parameterization becomes greater with increasing wind speed compared with the estimates of Soloviev et al. (2014). This suggests that using the new *C*_k_/*C*_d_ in operational models may help reduce the negative bias in TC intensity prediction for major hurricanes, as demonstrated in the next section.

## Effects of new *C*_k_/*C*_d_ parameterization on TC intensity prediction

**FIGURE 3:**
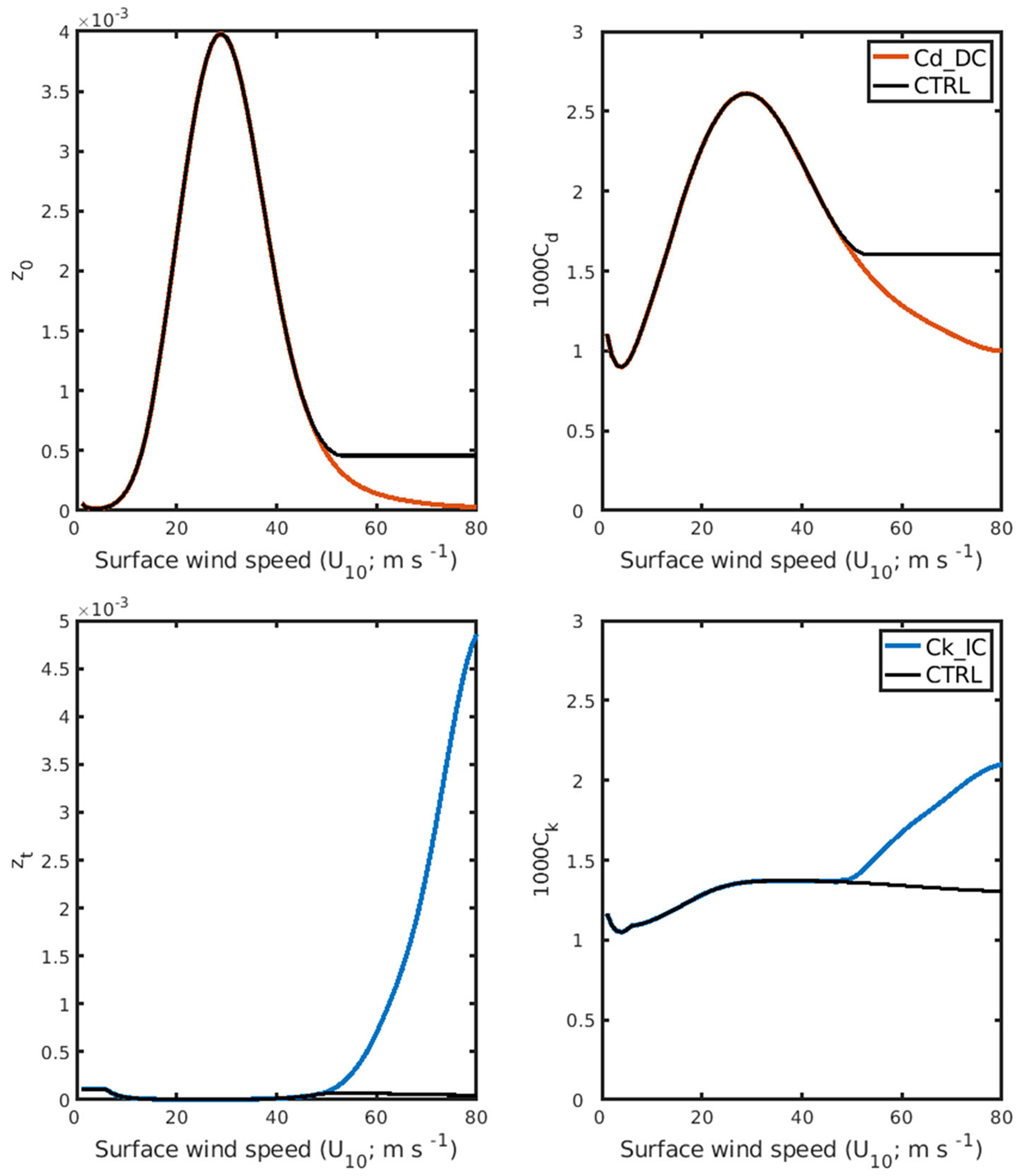
Parameterization of **(A)** roughness lengths for momentum z_*o*_, **(B)** drag coefficient C_d_, **(C)** roughness lengths for heat and humidity, and **(D)** heat exchange coefficient *C*_k_ as a function of 10-m wind speed, as used in the CTRL, Cd_DC and Ck_IC experiments (CTRL, black curve; Cd_DC, red curve; Ck_IC, blue curve).

To examine the impact of the new parameterization of enthalpy-momentum exchange coefficients, we perform three sets of numerical experiments using the HWRF. In the first experiment (CTRL), we use the original *C*_d_ parameterization of the HWRF (version 3.9a), in which *C*_d_ and the surface roughness lengths (*z_o_*) level off at 50 m s^−1^ (black line in Figures 3A, B). This is based on Soloviev et al. (2014) with slight modifications (Biswas et al., 2018). The other two experiments employ the increasing *C*_k_/*C*_d_ trend derived from our *T*_80_-based parameterization. The increasing *C*_k_/*C*_d_ ratio can mean that *C*_d_ decreases, or *C*_k_ increases, or both *C*_d_ decreases and *C*_k_ increases. It is difficult to modify both *C*_d_ and *C*_k_ simultaneously because we cannot deduce the ratio accurately, and limited computation resources are not enough to test every possible combination of *C*_k_ and *C*_d_. Therefore, to investigate the individual impact of the coefficients on TC intensification, we employ two types of increasing *C*_k_/*C*_d_ parameterization: 1) decreasing *C*_d_ with nearly constant *C*_k_ (Cd_DC, red line in Figures 3A, B; CTRL, black line in Figures 3C, D) and 2) increasing *C*_k_ with the original *C*_d_ (Ck_IC, blue line in Figures 3C, D; CTRL, black line in Figures 3A, B).

**FIGURE 4:**
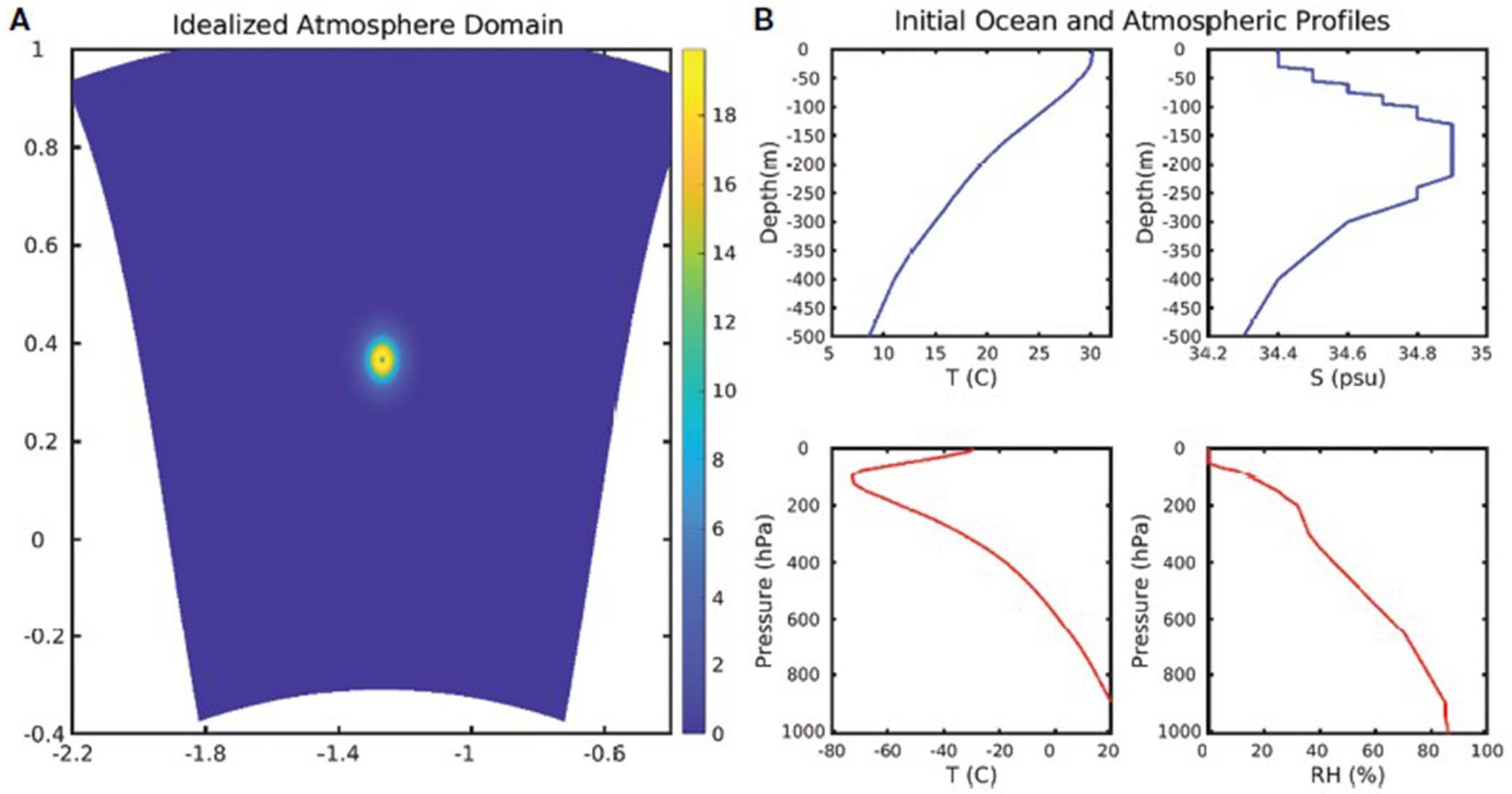
**(A)** Model domain for idealized experiments. Colors show wind speed with the initial vortex situated at the center of the domain. **(B)** Initial oceanic and atmospheric profiles used in idealized experiments. All initial temperature and salinity profiles for the ocean and initial temperature and humidity sounding are horizontally uniform.

The effects of the modified flux parameterizations on TC simulations are investigated using three sets of experiments—CTRL, Cd_DC, and Ck_IC (Table 1)—for idealized and real TC cases, in which all experiments are identical except that different surface exchange coefficients are used. We first investigate the sensitivity of parameterization in an idealized uncoupled experiment where the atmospheric model receives no feedback from the ocean. The experiment was conducted using an idealized HWRF framework configured for the operational HWRF triple domain configuration with a grid spacing of 13.5-, 4.5-, and 1.5 km (Biswas et al., 2018). The initial intensity of the idealized vortex was 20 m s^−1^ and the radius of maximum winds was 90 km, which is embedded in a quiescent ambient. The base-state temperature and humidity profile are based on Jordan’s Caribbean sounding (Gray et al., 1975). The SST is constant in time and space (304.75 K), and there is no land in the domain (Figure 4A).

Next, a series of idealized coupled TC simulations are conducted to investigate how the new *C*_k_/*C*_d_ parameterization under high winds modulates the air-sea interactions and how it affects TC intensification. A three-dimensional ocean model, Message Passing Interface Princeton Ocean Model-Tropical Cyclone (MPIPOM-TC), is embedded into the HWRF (HWRF-MPIPOM-TC). A coupler developed by NCEP serves as a hub for MPI communications between the HWRF atmosphere and MPIPOM-TC by which the surface fluxes and SSTs are exchanged between the HWRF atmospheric grids and the MPIPOM-TC grids. The initial ocean fields used for this experiment are horizontally uniform and based on temperature and salinity profiles as shown in Figure 4B.

In addition to the idealized experiments, two real-case experiments for Hurricane Irma (2017) and Typhoon Mangkhut (2018) are conducted. In this experiment, as in the idealized cases, the three parameterizations in Table 1 are also tested using a coupled model. Each simulation is integrated for 126 hours, and the output is saved every three hour. As described earlier, the three sets of experiments are identical except for using different surface exchange coefficients.

**FIGURE 5:**
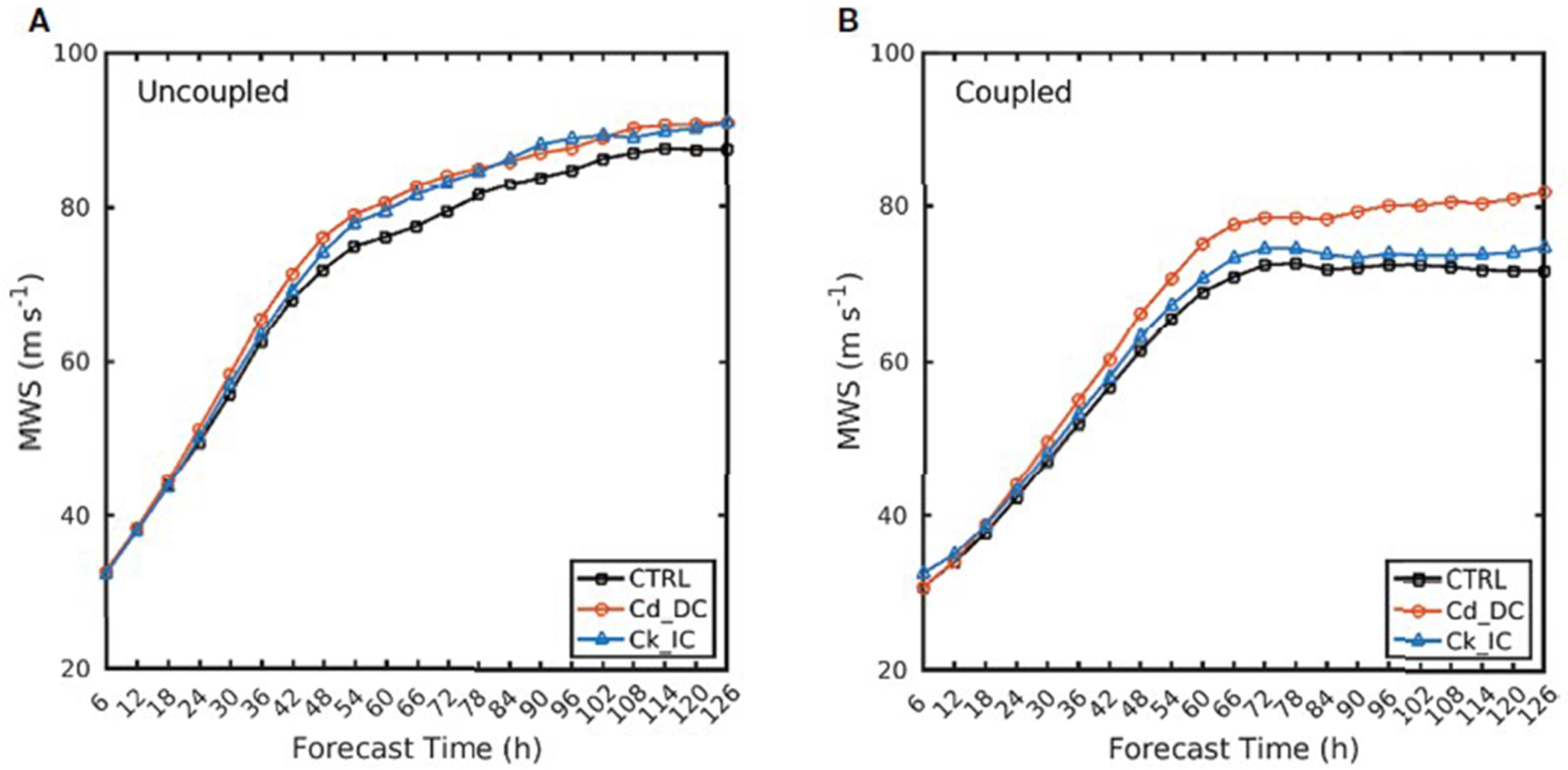
Time series of maximum wind speed (m s ^−1^) for three simulations of idealized uncoupled **(A)** and coupled **(B)** experiments. Wind speeds are smoothed by a 48-h moving average.

Figure 5 shows the time series of 48-h moving averaged MWS from three experiments in both idealized uncoupled and coupled experiments. Here, the TC intensity changes for the three different parameterizations have a similar trend during the first 24 h when the MWS has not yet reached 50 m s^−1^. This is because the *C*_d_ and *C*_k_ parameterizations at wind speeds below 50 m s^−1^ are the same in all three experiments. However, after 24 h, the Cd_DC and Ck_IC simulations persistently produce a greater MWS than the CTRL simulation. The TC intensity in the coupled model is lower in the uncoupled experiment due to the negative feedback from the ocean. Interestingly, in uncoupled simulations, the MWS in the Cd_DC and Ck_IC are similar to each other at all forecast times (Figure 5A), while, in coupled experiments, Cd_DC simulates a stronger than Ck_IC by up to 14 m s^−1^ (Figure 5B). To examine why the intensity differs between the Cd_DC and Ck_IC in coupled experiments, we conducted six idealized ensemble experiments forced with different combinations of atmospheric initial and boundary conditions and analyzed TC-induced sea surface temperature cooling (SSC) as well as air-sea humidity difference *(ΔQ* = *Q_s_* - *Q_a_)*. The SSC is calculated as the SST of each forecast time from 6 h to 120 h minus the SST of the initial forecast time, in which the SST is averaged within 50 km from the TC center for each forecast time.

**FIGURE 6:**
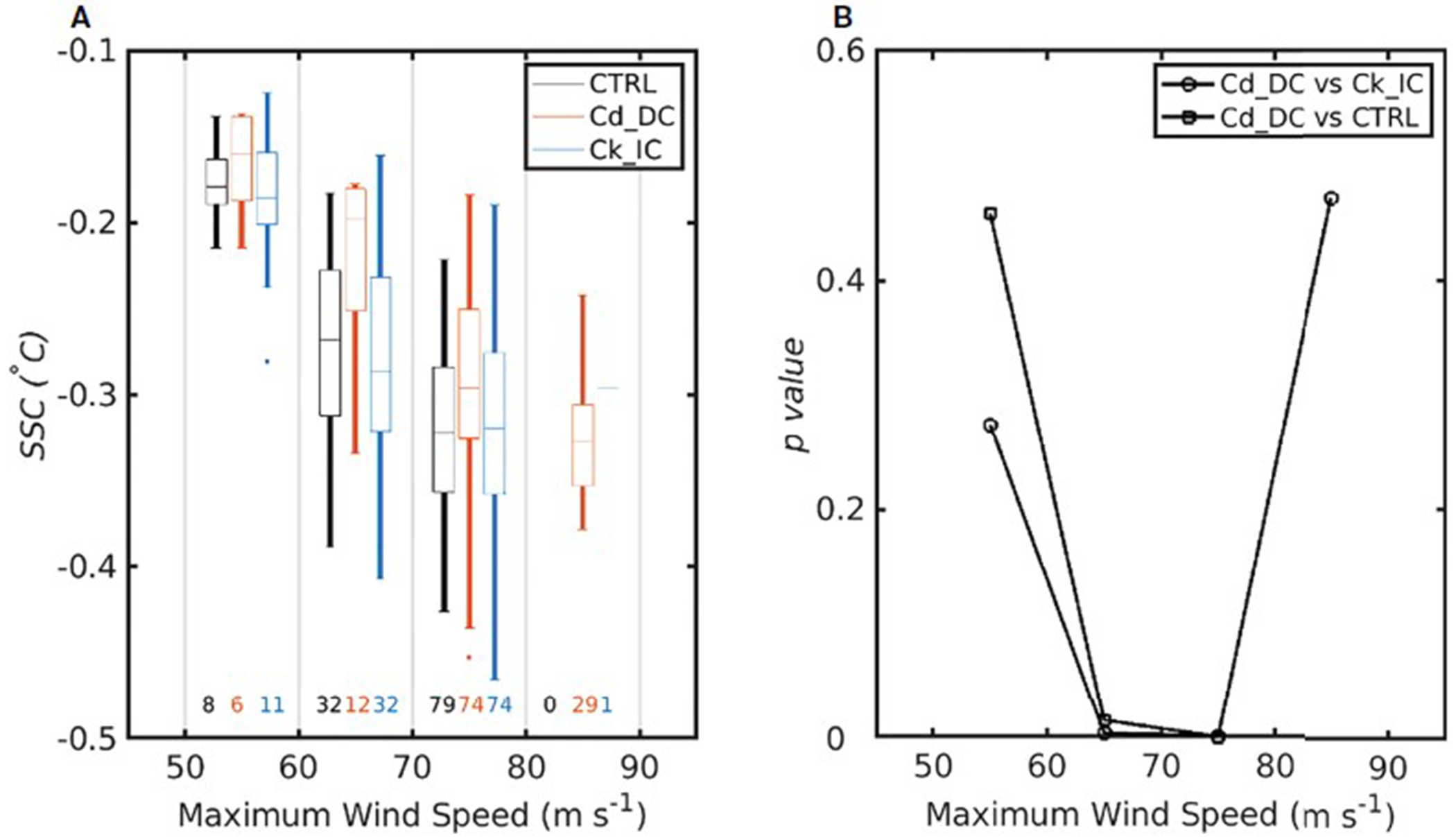
**(A)** Boxplots for sea surface cooling (°C) as a function of MWS at each 10-m s^−1^ interval. The lines denote the median values, and the box covers the 25%-75% quantile. The bars show the minimum and maximum values, and the points show the outliers. Colors indicate results for CTRL (black), Cd_DC (red) and Ck_IC (blue). The numbers above the x-axis denote the assigned TC case number for each experiment. **(B)** The distribution of *p*-value for the SSC differences between Cd_DC and Ck_IC (solid open circle) and between Cd_DC and CTRL (solid open square). The *p*-values are based on Student t-test.

**FIGURE 7:**
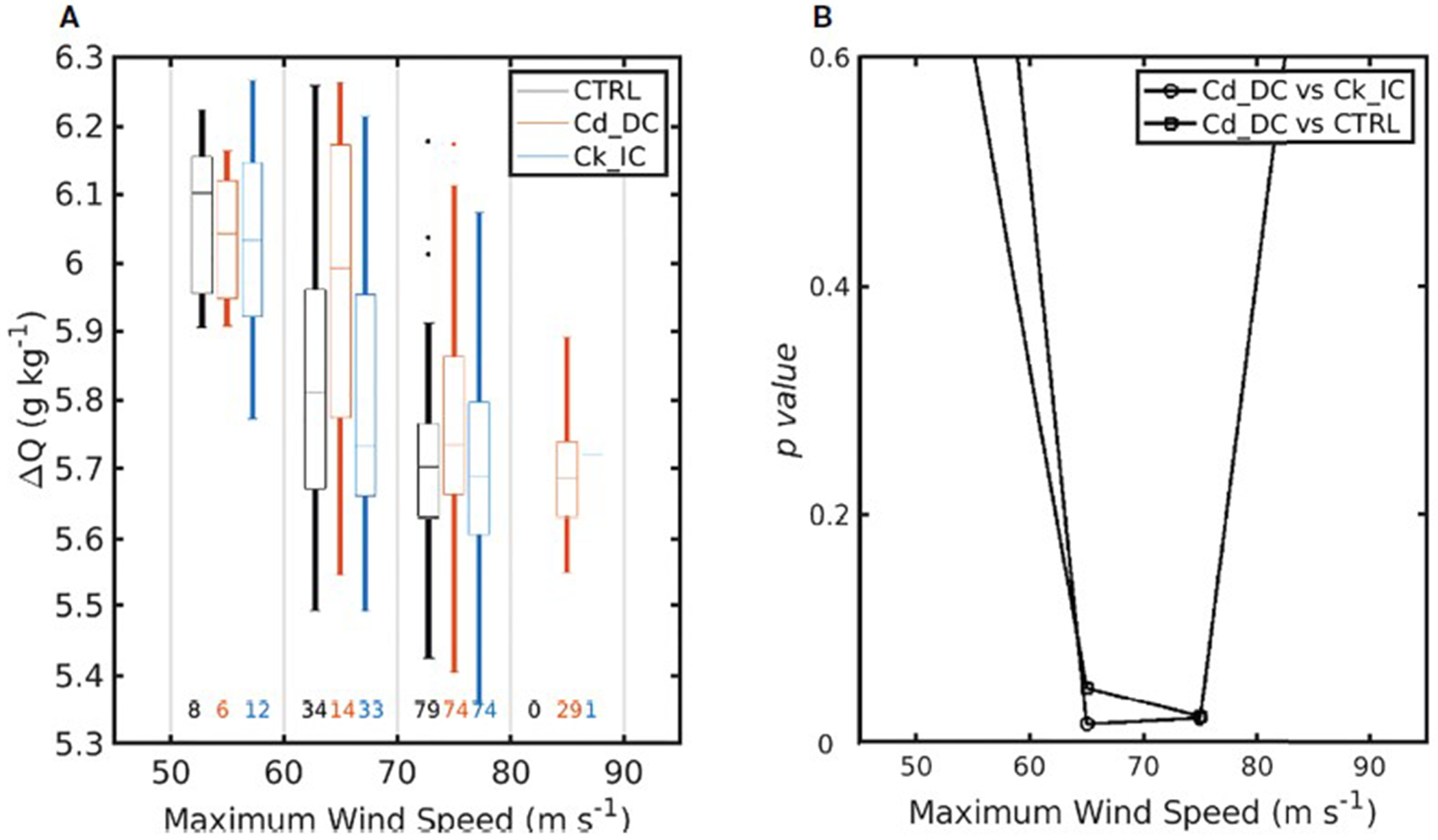
Same in Figure 6, but for **(A)** the air-sea moisture difference and **(B)** its p-values. The p in the p-values is italicization.

The SSC for all three experiments tends to increase as the MWS increases. A closer look reveals that the SSC from both CTRL and Ck_IC is greater than that of Cd_DC (particularly, the difference is statistically significant above the 99% confidence level between 60 m s^−1^ and 80 m s^−1^) (Figure 6). Because the wind stress is proportional to the C_*d*_ times the wind speed squared, a decreased *C*_d_ reduces the momentum flux into the ocean, inhibiting vertical mixing of the upper ocean. Consequently, SSC in the Cd_DC experiment is reduced, thereby increasing heat fluxes that contribute positively to TC intensification (Figure 7). Based on the bulk aerodynamic formula, increased C_*k*_ also contributes to the increase of heat fluxes favoring for TC intensification. Both decreased C_*d*_ and increased C_*k*_ under high winds can positively contribute to the TC intensification; however, the contribution of C_*d*_ is more significant than that of C_*k*_ because C*_d_* affects not only frictional dissipation but also the heat flux in high winds.

**FIGURE 8:**
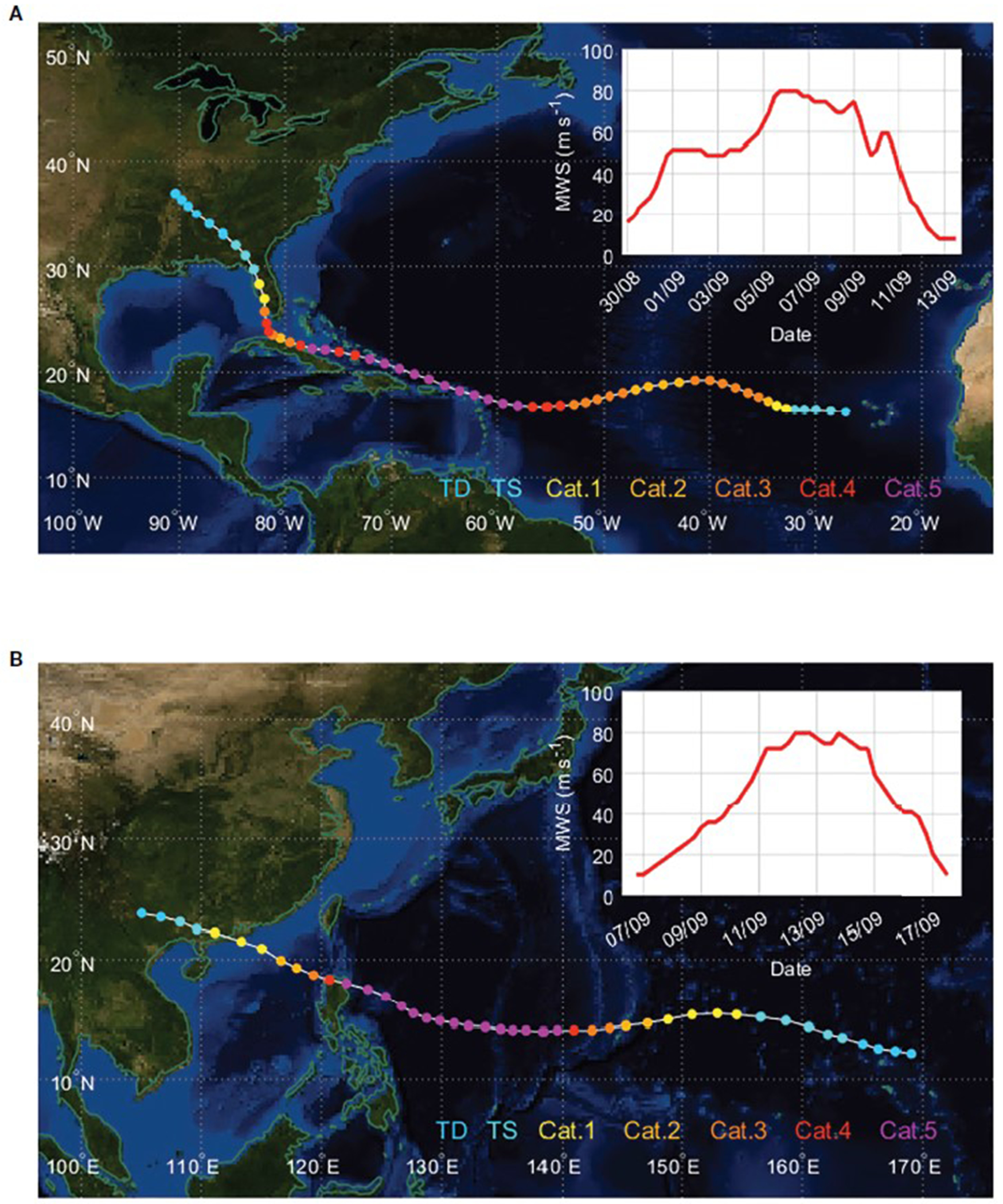
**(A)** Hurricane Irma over North Atlantic in 2017 and **(B)** Typhoon Mangkhut over the western North Pacific in 2018. The best-track positions and maximum wind speeds (in the box) are shown at 6-h intervals along with the intensity of the Saffir-Simpson wind scale (filled circle).

**FIGURE 9:**
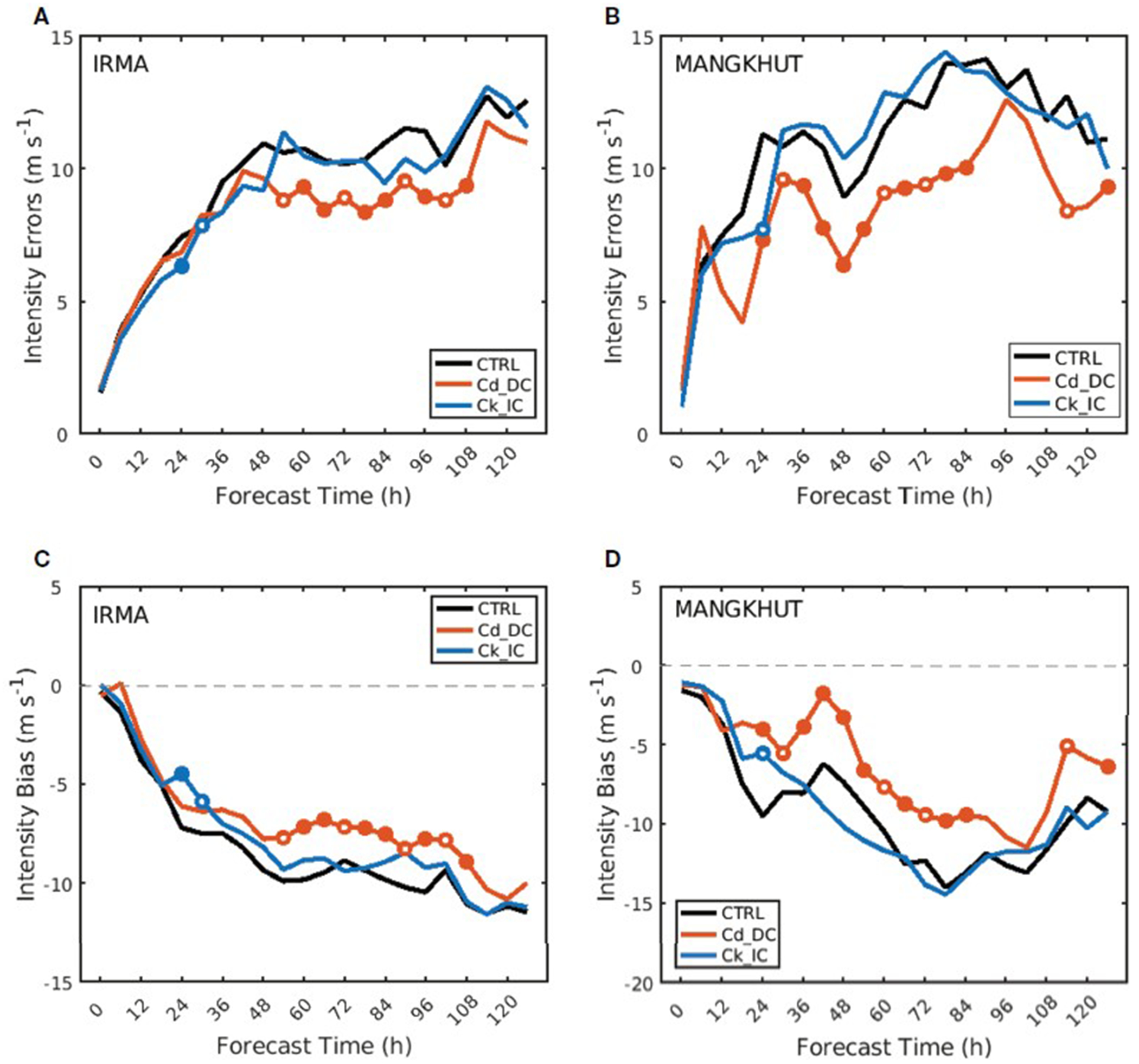
Comparisons of mean absolute error **(A, B)** and mean bias **(C, D)** against forecast lead time for three experiments, CTRL (black line), Cd_DC (red line), and Ck_IC (blue line), for Irma **(A, C)** and Mangkhut **(B, D)**. Red (blue) filled [open] circles indicate that the binned value for Cd_DC (Ck_IC) is significantly improved than for CTRL at the 99% [95%] confidence level (Student’s *t*-test).

To investigate the effectiveness of the proposed parameterization, Cd_DC, CK_IC, CTRL experiments are conducted for two Category 5 TCs: Hurricane Irma (Figure 8A) and Typhoon Mangkhut (Figure 8B). We forecasted 25 (15) times for Irma (Mangkhut) every 6 hours from 1200 UTC 30 August (0000 UTC 8 September) to 1200 UTC 5 September 2017 (1200 UTC 11 September 2018). The prediction results are evaluated for TC intensity (i.e., MWS) above 50 m s^−1^ with different parameterizations of the surface exchange coefficient in three experiments. Statistics show that Cd_DC outperforms both CTRL and Ck_IC in terms of the mean absolute error and bias in the intensity prediction of Irma and Mangkhut (Figure 9). For Irma, all absolute errors increase rapidly with the forecast lead time up to 48 h, but they level off thereafter. The difference between the three experiments becomes evident after 48 h, in which the error of Cd_DC is smaller by about 15% than that of CTRL after 48 h (Figure 9A). The bias differences between the experiments can mainly explain the reduction in error. That is, the negative bias of Cd_DC is smaller by 19% than that of CTRL after 48 h (Figure 9C). In contrast to Cd_DC, Ck_IC errors are slightly lower or similar to the CTRL errors. For Ck_IC, the mean absolute error after 48 h is reduced by about 3% compared to the CTRL. For the case of Mangkhut, the bias and errors of Cd_DC are significantly reduced at most forecast lead times (Figures 9B, D). In particular, for all forecast leads, Cd_DC reduced the absolute error on average by ~32% compared to CTRL. On the other hand, Ck_IC forecasts are not significantly improved compared to CTRL. These results are similar to those of Irma, implying that decreasing C_*d*_ can reduce the TC intensity error more effectively than increasing C*_k_* under high winds.

In the Cd_DC experiment, the constant *C*_d_ at winds above 50 m s^−1^ is only replaced by a *C*_d_ that continues to decrease with increasing wind speed. This suggests that such improvement in intensity prediction for the two TCs is attributed to the reduction in *C*_d_. To summarize, the decrease in *C*_d_ at high winds provided two favorable conditions for TC intensification: (1) a decrease in frictional dissipation at the air-sea interface and (2) and an increase of air-sea enthalpy (latent plus sensible heat) fluxes due to the reduced SST cooling caused by the decrease in momentum flux into the sea. Again, this emphasizes that decreasing *C*_d_ at high winds might be vital in improving intensity prediction for major TCs.

## Conclusions and discussions

We present a new parameterization of *C*_k_/*C*_d_ at high wind speeds above 40 m s^−1^ that uses the relationship between the observed LMI and theoretical MPI. This new parameterization shows that *C*_k_/*C*_d_ increases with wind speed. We conduct triplet numerical experiments using the HWRF model, called CTRL, Cd_DC, and Ck_IC, to investigate whether the proposed parameterization can improve TC intensity prediction. The first uses *C*_d_ that is constant at wind speeds above 50 m s^−1^, the second uses *C*_d_ that decreases continuously with wind speed (as *C*_d_ decreases, *C*_k_/*C*_d_ increases with wind speed due to the constant *C*_k_ used in the HWRF model) and the last uses *C*_k_ that increases continuously with wind speed (as *C*_k_ increases, *C*_k_/*C*_d_ increases with wind speed due to the nearly constant *C*_d_ above 50 m s^−1^ used in the HWRF model). Ck_IC and Cd_DC show stronger TC intensity than CTRL in both idealized uncoupled and coupled experiments. However, there is no significant difference in TC intensity between Ck_IC and Cd_DC in the uncoupled simulations, while the intensity of Cd_DC appears to be stronger than Ck_IC in the coupled simulations. The results show that the decreased *C*_d_ not only reduces frictional dissipation but also causes a reduction in the air-sea momentum flux, consequently inhibiting SSC. The extra energy supplied by the reduced SSC in the Cd_DC experiment simulated a stronger TC than in the CK_IC experiment. Similar results are found in the numerical simulations of two category-5 TCs, Hurricane Irma (2017) and Typhoon Mangkhut (2018). Both Cd_DC and Ck_IC reduced negative bias in TC intensity prediction; however, only the former result was statistically significant. This suggests that decreasing *C*_d_ may be more effective than increasing *C*_k_ in reducing the underestimation of TC intensity in the coupled model simulation.

Efforts have been made in recent years to find the optimal parameterization of air-sea exchange coefficients on TC evolution using an atmosphere-ocean coupled modeling system (Chen et al., 2018; Liu et al., 2022). Using the atmosphere-ocean coupled experiment, Chen et al. (2018) performed a sensitivity test of three *C*_d_ parameterizations (increase, decrease, and level off in high winds) for Hurricane Katrina. Their study indicated that the use of a momentum flux parameterization with decreasing *C*_d_ and default *C*_k_ (Chen and Yu, 2016; *C*_k_/*C*_d_ ratio increased) in high winds improve the accuracy of TC intensity prediction for very strong wind. Liu et al. (2022) performed sensitivity experiments of *C*_d_ parameterization using a coupled model and compared the sea surface cooling reproduced in the model with buoy data. They found that the heat flux was significantly affected by the *C*_d_-induced sea surface cooling effect rather than the change in wind speed in the model results. Their main findings are consistent with ours regarding the importance of the use an atmosphere-ocean coupled model, resulting in better understanding of the flux exchange between TCs and the ocean.

In this study, we estimate *C*_k_/*C*_d_ under the most intense TCs using the MPI approach, which has some limitations that need to be discussed. First, technically, the MPI defined by Equation 1 is the axisymmetric gradient wind at the top of the boundary layer, while the LMI is the maximum 1-minute sustained 10 m surface wind, which is a different metric from the MPI (Emanuel and Rotunno, 2011). This study uses a VREDUC factor of 0.8 in the MPI calculation using Emanuel’s ‘pcmin’ code to reduce the maximum gradient wind (Vmax) to the surface wind. However, a possible scenario is that *C*_k_/*C*_d_ is constant, but the mixing of momentum in the boundary layer changes as a function of the intensity such that VREDUC is variable instead of constant in a comparison of MPI and LMI. This is based on the relationship between *C*_k_/*C*_d_ and Vmax, which can be affected by the parameterization of turbulence in an axisymmetric model (Bryan and Rotunno, 2009). If the identified relationship is actually between the gradient wind and surface wind as a function of wind speed, both 40 m s^−1^ and 80 m s^−1^ surface winds should have the same gradient wind as that of 80 m s^−1^, since our regression function of *C*_k_/*C*_d_ (blue line in Figure 2) shows ~0.5 at 40 m s^−1^ and ~1.0 at 80 m s^−1^. This cannot happen in reality, as the TC should keep a similar magnitude of the relationship between the gradient wind at the top of the boundary layer and the surface wind.

Second, when the VREDUC factor of 0.8 reduces the gradient wind to the surface wind, the reduced values are not constant; they are a function of wind speed. The factor reduces 80 m s^−1^ to 64 m s^−1^ (16 m s^−1^ reduction) and 40 m s^−1^ to 32 m s^−1^ (8 m s^−1^ reduction, which is half the reduction at 80 m s^−1^). Actually, the turbulence length scale in an axisymmetric model may affect the relationship between *C*_k_/*C*_d_ and Vmax (Bryan and Rotunno, 2009; Bryan, 2012), however not imply that the turbulence length scale is a function of wind speed. There may also be concerns about going from the axisymmetric wavenumber (0 Vmax) to the Earth-relative wind anywhere in the storm. However, we believe that the problem of going from axisymmetric to point values would be small, given that we chose only storms that moved relatively slowly. Vukicevic et al. (2014) also showed that the azimuthal wavenumber 0 + 1 wind strongly correlates with maximum intensity (as traditionally defined), providing additional support for our approximation.

Lastly, based on theory, the *k* in Equation 1 is the 10 m enthalpy at the radius of maximum winds (RMW), not the ambient enthalpy. However, it is technically challenging to calculate an accurate enthalpy at the RMW because the spatial resolution (1.5° × 1.5°) of the MERRA data used in the present analysis is not enough to resolve the eyewall and the uncertainty in the estimated RMW. Therefore, this study calculated the enthalpy and MPI by averaging values within a 300 km radius of the storm center rather than using point values at the RMW. We also estimated the MPI using prestorm conditions (3 days before LMI) (Lin et al., 2013) to avoid modifications in the atmospheric and oceanic profiles due to air and moisture supply and oceanic mixing accompanied by TCs. A series of sensitivity experiments revealed that the averaging areas (100 km to 500 km or a donut-shaped path along the RMW) and the MPI calculation time (0 to 3 days before MPI) do not significantly affect the main results.

There are reasonably convincing studies for decreasing *C*_d_ and increasing *C*_k_ under severe wind. As wind speed increases, the ocean becomes covered by foam and *C*_d_ is reduced due to the slippery surface after the foam at the air-sea interface disappeared (Powell et al., 2003). Donelan (2018) showed that *C*_d_ decreased due to the sheltering of the surface in the lee of steep waves. Soloviev et al. (2014) suggest that *C*_d_ can also decrease because the Kelvin-Helmholtz instability at the air-sea interface leads to an absence of short surface waves (the instability grows more quickly due to large shear for short waves). It is known that sea spray is a crucial factor in the development of TCs, which are responsible for the enhancement of energy flux from the ocean to the atmosphere (Andreas and Emanuel, 2001; Andreas, 2011). Andreas (2011) projected a significant *C*_k_ increase with wind speed because of the increasing importance of spray-mediated transfer although the *C*_k_ ratio is varied with surface temperature and atmosphere stratification.

An accurate estimation of air-sea enthalpy and momentum fluxes is key to improving intensity forecast accuracy in numerical weather prediction models (Sroka and Emanuel, 2021). Whether *C*_k_/*C*_d_ decreases or increases at extreme winds above 50 m s^−1^ remains debatable. Limitations also exist in our method of estimating *C*_k_/*C*_d_. Nevertheless, this study provides important clues for understanding *C*_k_/*C*_d_ behavior at high winds, where field observations are difficult. We also show through real TCs simulations that applying the new *C*_k_/*C*_d_ parameterization to TC prediction can significantly reduce the negative intensity bias. However, numerical experiments and verifications in more cases are needed to generalize the present results. Our prediction results still show negative biases in high winds, suggesting that other factors, such as TC model resolutions and physics should be further improved.

## Supplementary Material

Table S1

Figures S1-S3

The Supplementary Material for this article can be found online at: https://www.frontiersin.org/articles/10.3389/fmars.2022.1046511/full#supplementary-material

## Figures and Tables

**TABLE 1:** Numerical experimental design to investigate the effect of air-sea flux parameterizations on TC simulations using HWRF.

Experimental Name	Air-sea flux parameterizations above 50 m s^−1^	
	C_d_	C_k_
CTRL	Original HWRF (constant)	Original HWRF (nearly constant)
Cd_DC	Decreasing C_d_	Original HWRF (nearly constant)
Ck_IC	Original HWRF (constant)	Increasing C_k_

## Data Availability

The original contributions presented in the study are included in the article/Supplementary Material. Further inquiries can be directed to the corresponding author.

## Appendix

The new formulation for z_*o*_ a used in the Cd_DC experiment is

$$Z_{o}=\exp\left(p10+p11\times W+p12\times W^{2}+p13\times W^{3}\right),\;W\;\leq 6.5\;m\;s^{-1}$$

$$Z_{o}=p25\times W^{5}+p24\times W^{4}+p23\times W^{3}+p22\times W^{2}+p21\;\times W+p20,\;6.5\;m\;s^{-1}\;<W\leq 15.7\;m\;s^{-1}$$

$$Z_{o}=\exp(p35\times W^{5}+p34\times W^{4}+p33\times W^{3}+p32\times W^{2}\;+p31\times W+p30,\;15.7\;m\;s^{-1}\;\;<W\leq 46.0\;m\;s^{-1}$$

$$Z_{o}=10\;\times \left(\frac{1}{\exp\sqrt{\frac{0.16}{p^{45\times W^{5}+p44\times W^{4}+p43\times W^{3}+p42\times W^{2}+p41\times W+p40}}}}\right),W\;>46.0\;m\;s^{-1}$$

where W (m s^−1^) is the wind speed, p10 = −8.396975715683501e+00, p11 = −1.597898515251717e+00, p12 = 2.855780863283819e-01, p13 = −1.296521881682694e-02, p20 = 2.147264020369413e-05, p21 = 1.739759082358234e-07, p22 = −1.240239171056262e-06, p23 = 1.962282433562894e-07, p24 = 3.281964357650687e-09, p25 = 3.790846746036765e-10, p30 = −1.663993561652530e+01, p31 = 1.255457892775006e+00, p32 = −6.139315534216305e-02, p33 = 1.735308193700643e-03, p34 = −2.793849676757154e-05, p35 = 1.840430200185075e-07, p40= −0.016943180834872e+00, p41 = 0.001980799375510e+00, p42= −7.499441958611604e-05, p43 = 1.330088992305179e-06, p44= −1.138431432537254e-08, p45 = 3.806734376022113e-11
