# Supplementary material for: New parameterization of air-sea exchange coefficients and its impact on intensity prediction under major tropical cyclones
Source: Front Mar Sci. Author manuscript; Available in PMC 2025 Aug 16. (PMC12355542; doi:10.3389/fmars.2022.1046511)
Supplement: Table S1 [file NIHMS2100040-supplement-Table_S1.docx]

Table S1 TC, period, maximum sustained wind speed (kt), minimum central pressure (hPa), and the Saffir-Simpson wind scale for 84 selected TCs over the WNP, ENP, and NA basins over 1980–2015.

| Basin | Year | Storm |
| --- | --- | --- |
| AL  (8) | 1995 | LUIS (13L) |
|  | 1996 | EDOUARD (05L) |
|  | 1996 | FRAN (06L) |
|  | 1996 | HORTENSE (08L) |
|  | 1997 | ERIKA (07L) |
|  | 2001 | FELIX (07L) |
|  | 2003 | JUAN (15L) |
|  | 2010 | IGOR (11L) |
| EP  (30) | 1982 | JOHN (10E) |
|  | 1982 | NORMAN (14E) |
|  | 1982 | SERGIO (18E) |
|  | 1983 | ADOLPH (01E) |
|  | 1984 | FAUSTO (06E) |
|  | 1984 | POLO (17E) |
|  | 1985 | TERRY (19E) |
|  | 1987 | OTIS (15E) |
|  | 1987 | RAMON (17E) |
|  | 1988 | LANE (12E) |
|  | 1990 | JULIO (13E) |
|  | 1990 | MARIE (16E) |
|  | 1991 | JIMENA (12E) |
|  | 1991 | KEVIN (13E) |
|  | 1992 | CELIA (04E) |
|  | 1992 | FRANK (07E) |
|  | 1993 | GREG (08E) |
|  | 1993 | HILARY (09E) |
|  | 1994 | EMILIA (05E) |
|  | 1997 | NORA (16E) |
|  | 1998 | HOWARD (09E) |
|  | 1999 | EUGENE (08E) |
|  | 2000 | ALETTA (01E) |
|  | 2001 | GIL (08E) |
|  | 2001 | JULIETTE (11E) |
|  | 2002 | DOUGLAS (05E) |
|  | 2005 | KENNETH (11E) |
|  | 2006 | DANIEL (05E) |
|  | 2006 | HECTOR (09E) |
|  | 2010 | DARBY (05E) |
| WP  (46) | 1980 | DINAH (27W) |
|  | 1982 | PAT (04W) |
|  | 1982 | BESS (11W) |
|  | 1982 | IRVING (18W) |
|  | 1982 | JUDY (19W) |
|  | 1982 | KEN (20W) |
|  | 1982 | MAC (23W) |
|  | 1986 | BEN (16W) |
|  | 1987 | THELMA (05W) |
|  | 1987 | FREDA (13W) |
|  | 1987 | IAN (17W) |
|  | 1988 | HAL (14W) |
|  | 1988 | NELSON (20W) |
|  | 1988 | ODESSA (21W) |
|  | 1990 | MIKE (27W) |
|  | 1990 | RUSS (31W) |
|  | 1991 | WALT (04W) |
|  | 1991 | PAT (24W) |
|  | 1991 | SETH (26W) |
|  | 1992 | COLLEEN (26W) |
|  | 1993 | ROBYN (13W) |
|  | 1994 | WALT (10W) |
|  | 1994 | WILDA (35W) |
|  | 1995 | OSCAR (17W) |
|  | 1995 | WARD (26W) |
|  | 1995 | ZACK (28W) |
|  | 1996 | KIRK (13W) |
|  | 1996 | YATES (28W) |
|  | 1997 | WINNIE (14W) |
|  | 2000 | KIROGI (05W) |
|  | 2001 | KONG-REY (09W) |
|  | 2001 | MAN-YI (12W) |
|  | 2001 | NARI (20W) |
|  | 2001 | KROSA (24W) |
|  | 2002 | RAMMASUN (09W) |
|  | 2002 | FENGSHEN (12W) |
|  | 2002 | RUSA (21W) |
|  | 2004 | TINGTING (11W) |
|  | 2005 | TALIM (13W) |
|  | 2006 | CHANCHU (02W) |
|  | 2007 | MAN-YI (04W) |
|  | 2008 | NAKRI (06W) |
|  | 2010 | CHABA (16W) |
|  | 2012 | TEMBIN (15W) |
|  | 2012 | JELAWAT (18W) |
|  | 2013 | FRANCISCO (26W) |
