# Supplementary material for: New parameterization of air-sea exchange coefficients and its impact on intensity prediction under major tropical cyclones
Source: Front Mar Sci. Author manuscript; Available in PMC 2025 Aug 16. (PMC12355542; doi:10.3389/fmars.2022.1046511)
Supplement: Figures S1-S3 [file NIHMS2100040-supplement-Figures_S1-S3.docx]

**Appendix A**

New parameterization of air-sea exchange coefficients and its impact on intensity prediction under major tropical cyclones

Woojeong Lee^1,2†^, Sung-Hun Kim^3†^, Il-Ju Moon^2*^, Michael M. Bell^4^, and Isaac Ginis^5^

^1^Forecast Research Department, National Institute of Meteorological Sciences, 33 Seohobuk-ro, Seogwipo, Jeju, 63568, Republic of Korea

^2^Graduate Program in Marine Meteorology/Typhoon Research Center, Jeju National University, 102 Jejudaehak-ro, Jeju 63243, Republic of Korea

^3^Korea Institute of Ocean Science and Technology, 385 Haeyang, Yeongdo, Busan, 49111, Republic of Korea

^4^Department of Atmospheric Science, Colorado State University, Fort Collins, Colorado, USA

^5^Graduate School of Oceanography, University of Rhode Island, Narragansett, Rhode Island, USA

*Corresponding author: Il-Ju Moon, ijmoon@jejunu.ac.kr

^†^ These authors have contributed equally to this work and share first authorship


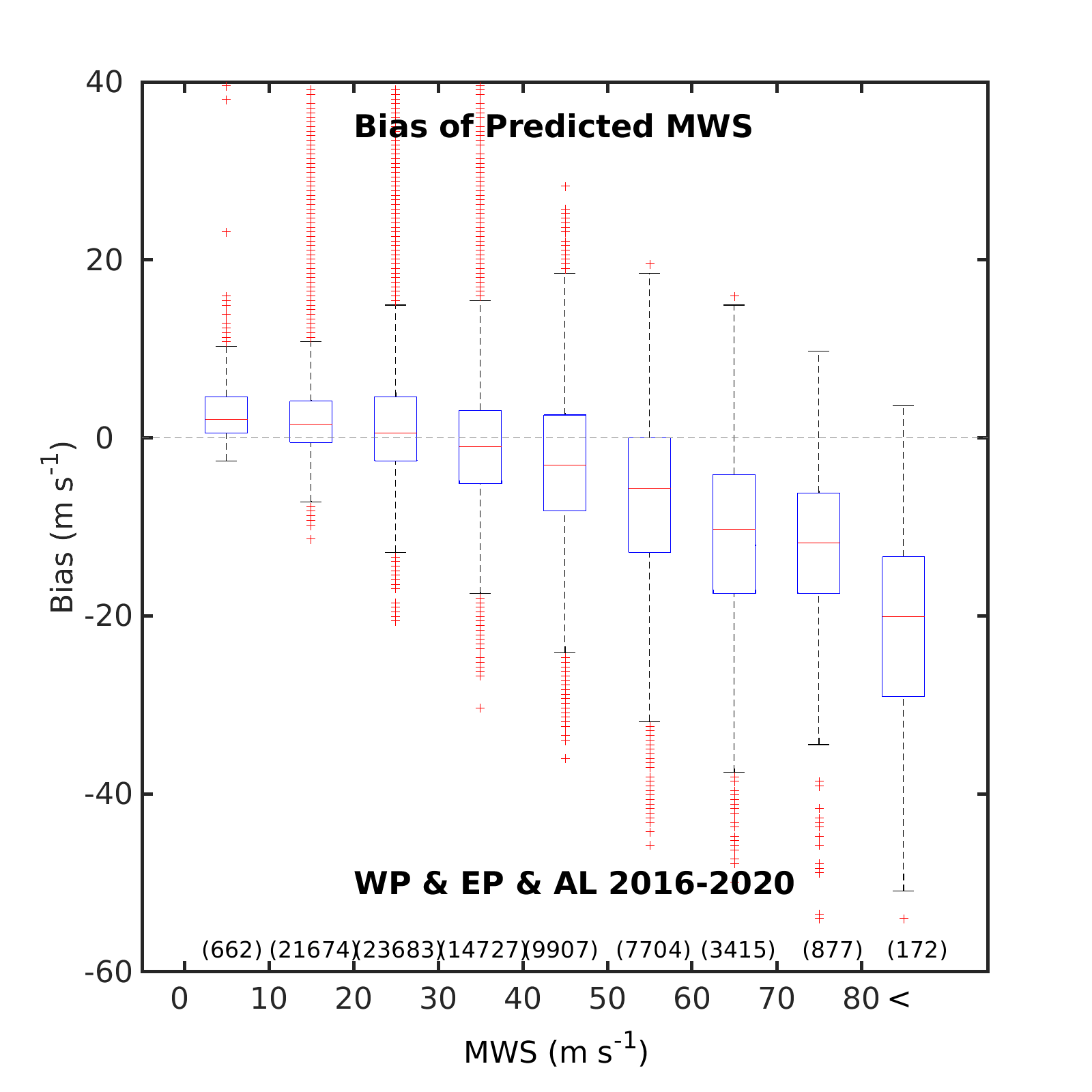


Fig. S1 Evaluation results of operational HWRF intensity predictions for 82,821 TC cases in 2016-2020 NA, ENP and WNP. The values represent the bias of the predicted MWS against that of the measured MWS. The candle bars indicate the medians (red horizontal bars) and their 95% confidence intervals (dashed bars) for biases within each 10 m s^−1^ interval of the MWS. The numbers in parentheses above the x axis denote the assigned TC case number.


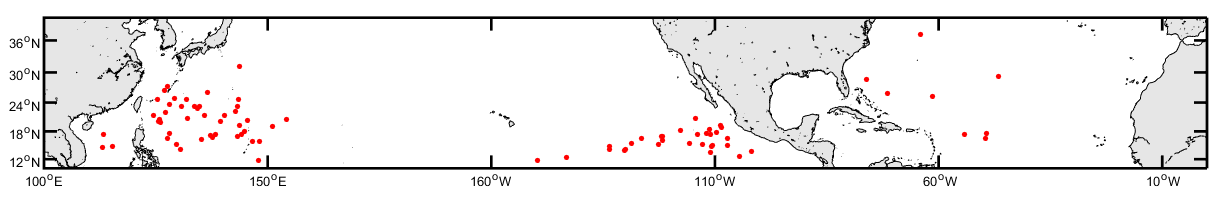


Fig. S2 Locations at the LMI for TCs (total 84) selected for the *C*_k_/*C*_d_ parameterization over the WNP, ENP, and NA basins over 1980–2015.

**
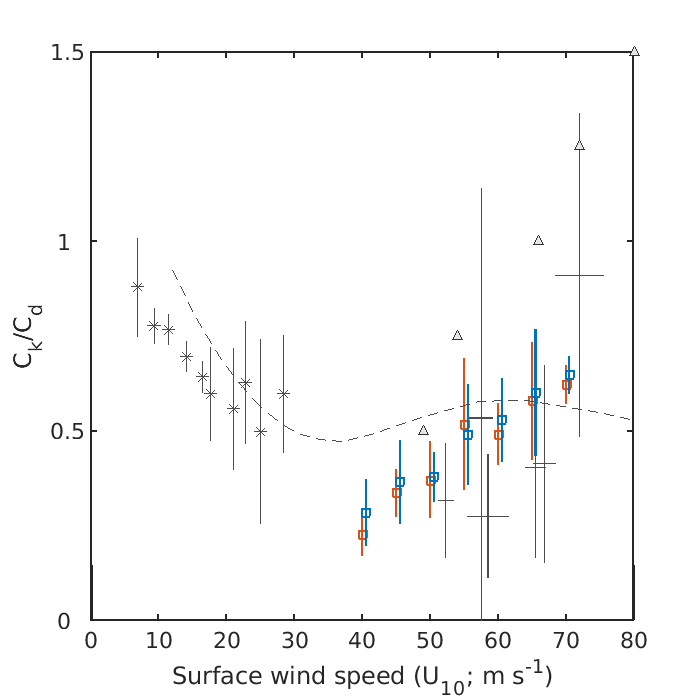
**

Fig. S3 Sensitivity of SST data to calculation of *C_k_*/*C_d_*. Comparison of *C*_k_/*C*_d_ estimated using prestorm SST from GHRSST (red) and GODAS (blue) against *U*_10_. The squares indicate the mean values of *C*_k_/*C*_d_ within each 5 m s^−1^ interval of wind speed. The error bars represent one standard deviation. The dashed gray lines indicate data adapted from Soloviev et al. (2014), and the gray triangles indicate data adapted from Emanuel (1995). The mean and 95% confidence intervals of diverse laboratory and measurement results (Bell et al., 2012; DeCosmo et al., 1996; Haus et al., 2010; Zhang et al., 2008) are shown in gray asterisks and solid lines.
